# Effects of Engineered *Saccharomyces cerevisiae* Fermenting Cellobiose through Low-Energy-Consuming Phosphorolytic Pathway in Simultaneous Saccharification and Fermentation

**DOI:** 10.4014/jmb.2111.11047

**Published:** 2021-12-09

**Authors:** Hyo-Jin Choi, Yong-Su Jin, Won-Heong Lee

**Affiliations:** 1 Department of Bioenergy Science and Technology, and Department of Integrative Food, Bioscience and Biotechnology, Chonnam National University, Gwangju 61186, Republic of Korea; 2 Department of Food Science and Human Nutrition, and Carl R. Woese Institute for Genomic Biology, University of Illinois at Urbana-Champaign, Urbana, IL 61801, USA

**Keywords:** Cellulosic ethanol, simultaneous saccharification and fermentation, mutant cellodextrin facilitator, cellobiose phosphorylase, engineered *Saccharomyces cerevisiae*

## Abstract

Until recently, four types of cellobiose-fermenting *Saccharomyces cerevisiae* strains have been developed by introduction of a cellobiose metabolic pathway based on either intracellular β-glucosidase (GH1-1) or cellobiose phosphorylase (CBP), along with either an energy-consuming active cellodextrin transporter (CDT-1) or a non-energy-consuming passive cellodextrin facilitator (CDT-2). In this study, the ethanol production performance of two cellobiose-fermenting *S. cerevisiae* strains expressing mutant CDT-2 (N306I) with GH1-1 or CBP were compared with two cellobiose-fermenting *S. cerevisiae* strains expressing mutant CDT-1 (F213L) with GH1-1 or CBP in the simultaneous saccharification and fermentation (SSF) of cellulose under various conditions. It was found that, regardless of the SSF conditions, the phosphorolytic cellobiose-fermenting *S. cerevisiae* expressing mutant CDT-2 with CBP showed the best ethanol production among the four strains. In addition, during SSF contaminated by lactic acid bacteria, the phosphorolytic cellobiose-fermenting *S. cerevisiae* expressing mutant CDT-2 with CBP showed the highest ethanol production and the lowest lactate formation compared with those of other strains, such as the hydrolytic cellobiose-fermenting *S. cerevisiae* expressing mutant CDT-1 with GH1-1, and the glucose-fermenting *S. cerevisiae* with extracellular β-glucosidase. These results suggest that the cellobiose-fermenting yeast strain exhibiting low energy consumption can enhance the efficiency of the SSF of cellulosic biomass.

## Introduction

To produce cellulosic biofuels efficiently, the development of *Saccharomyces cerevisiae*—a useful host for bioethanol production—to utilize intracellular cellobiose has been proposed as a strategy for avoiding glucose repression that frequently reduces product yield and productivity during the fermentation of mixed sugars derived from cellulosic biomass [1-5]. Indeed, engineered *S. cerevisiae*, which is capable of fermenting cellobiose, exhibits enhanced ethanol yield and productivity through simultaneous fermentation of cellobiose and other sugars compared with ethanol yield and productivity observed through sequential fermentation of glucose and other sugars [3, 6, 7]. In particular, *S. cerevisiae* strains fermenting cellobiose require lower amounts of cellulolytic enzymes and exhibit similar or even better ethanol production performance than those of the non-engineered strain (the parental *S. cerevisiae* fermenting glucose degraded from cellobiose by supplementation of extracellular β-glucosidase) during simultaneous saccharification and fermentation (SSF) of cellulose [8-10].

For *S. cerevisiae* to ferment cellobiose, it is essential to introduce genes encoding cellodextrin transporters (CDTs) and intracellular cellobiose degrading enzymes—components of the heterologous cellobiose metabolic pathway [11-13]. Previously, two types of cellodextrin transporters were identified from *Neurospora crassa*: energy-dependent CDT-1, an active transporter spending one mole of ATP for transporting one mole of cellobiose [11, 14]; and energy-independent CDT-2, a facilitator that does not spend ATP for cellobiose transport [11, 14]. Two types of intracellular cellobiose degrading enzymes have also been identified: intracellular β-glucosidase (GH1-1) from *Neurospora Crassa*, involved in the cellobiose hydrolysis requiring two moles of ATP to initiate glycolysis [11, 14]; and cellobiose phosphorylase (CBP) from *Saccharophagus degradans*, involved in the cellobiose phosphorolysis requiring only one mole of ATP to initiate glycolysis [12, 15]. On this basis, four types of the cellobiose-fermenting *S. cerevisiae* strains have been developed, as shown in Fig. S1. (1) hydrolytic cellobiose-fermenting *S. cerevisiae* with CDT-1 and GH1-1 (D-BT1 strain), (2) phosphorolytic cellobiose-fermenting *S. cerevisiae* with CDT-1 and CBP (D-CT1 strain), (3) hydrolytic cellobiose-fermenting *S. cerevisiae* with CDT-2 and GH1-1 (D-BT2 strain), and (4) phosphorolytic cellobiose-fermenting *S. cerevisiae* with CDT-2 and CBP (D-CT2 strain) [11, 12, 14, 15].

Although CDT-1 and CDT-2 exhibit similar affinity to cellobiose, CDT-1 demonstrates a two-fold higher cellobiose transporting activity than that of CDT-2 (V_max_ of CDT-1 ≈ 0.7 pmol/s vs. V_max_ of CDT-2 ≈ 0.35 pmol/s)[11]. The CBP-catalyzed reaction is thermodynamically unfavorable compared with the GH1-1-catalyzed reaction (ΔG^0^ of cellobiose phosphorolysis = 3.6 kJ/mol vs. ΔG^0^ of cellobiose hydrolysis = −12.5 kJ/mol) [12, 16, 17]. In addition, although CBP and GH1-1 exhibit almost the same V_max_, CBP demonstrates a two-fold lower affinity to cellobiose than that of GH1-1 (K_m_ of CBP = 0.65 mM vs. K_m_ of GH1-1 = 0.32 mM) [12]. In summary, the D-BT1 strain has been considered as the best cellobiose-fermenting yeast strain [12, 14, 15, 18]. Consequently, the priority of the cellobiose fermentation efficiencies of the cellobiose-fermenting yeast strains could be presented in the order of D-BT1 > D-CT1 ≈ D-BT2 > D-CT2.

However, the cellobiose fermentation performance of the phosphorolytic cellobiose-fermenting *S. cerevisiae* strains can be significantly improved by employing mutant CDTs, such as CDT-1 (F213L) and CDT-2 (N306I), which exhibit three-fold and six-fold enhanced cellobiose transporting activities than those of the wild-type CDT-1 and CDT-2, respectively [12, 15]. The phosphorolytic cellobiose-fermenting *S. cerevisiae* strains expressing either mutant CDT-1 or mutant CDT-2 (D-CT1m and D-CT2m strains) exhibited a similar fermentation performance compared with that of the hydrolytic cellobiose-fermenting *S. cerevisiae* expressing mutant CDT-1 (D-BT1m strain) and an even better fermentation performance than that of the hydrolytic cellobiose-fermenting *S. cerevisiae* expressing mutant CDT-2 (D-BT2m strain) in the cellobiose fermentation under micro-aerobic conditions [12, 15]. Consequently, the cellobiose fermentation efficiencies of the cellobiose-fermenting yeast strains with mutant transporters could be presented in the following order: D-BT1m ≈ D-CT1m ≈ D-CT2m > D-BT2m.

In the previous studies, in addition to cellobiose fermentation performance comparison, the ethanol production performance of the D-BT1m strain have been evaluated in the SSF of cellulose [8, 9]. Although both D-BT1m and D-CT1m strains showed superior ethanol production than that of the non-engineered *S. cerevisiae* strain fermenting glucose by extracellular supplementation of β-glucosidase [8, 9], the D-CT1m strain exhibited a slightly lower ethanol production compared with that of the D-BT1m strain, indicating that the energetic advantage of CBP was barely observed in the case of cellobiose-fermenting yeast strain expressing mutant CDT-1 during the SSF of cellulose [9].

This study aimed to evaluate whether the D-BT2m strain, expected to demonstrate an energy-saving effect compared with that of mutant CDT-1, could perform similar or better ethanol production in the SSF of cellulose compared with that of the D-BT1m strain. To this end, the ethanol production performance of D-BT2m and D-CT2m strains during SSF under various conditions were compared with that of D-BT1m and D-CT1m strains. In addition, the ethanol production of D-CT2m was compared with those of D-BT1m and the glucose-fermenting *S. cerevisiae* with extracellular β-glucosidase (D-56+188 strain) during SSF contaminated by lactic acid bacteria (LAB).

## Materials and Methods

### Strains, Plasmids, and Culture Conditions

*S. cerevisiae* D452-2 (*Matα*, *leu2*, *his3*, *ura3* and *can1*) [19] was used as the host strain for overexpression of the GH1-1 from *N. crassa* or the CBP from *Saccharophagus degradans* along with either the mutant active CDT-1 (F213L) or the mutant passive cellodextrin facilitator [CDT-2 (N306I)] from *N. crassa*. Plasmids pRS425-gh1-1, pRS425-CBP, pRS426-cdt1 (F213L), and pRS426-cdt2 (N306I) were previously constructed for overexpression of GH1-1, CBP, CDT-1 (F213L), and CDT-2 (N306I), respectively [11, 12, 15]. All *S. cerevisiae* strains and the plasmids used in this study are listed in Table 1.

Synthetic complete (SC) medium (6.7 g/l of yeast nitrogen base without amino acids, 0.625 g/l of complete supplement mixture without leucine and uracil, pH 6.0) containing 20 g/l of glucose, SCD20, was used for seed cultivation. Yeast extract-peptone (YP) medium (10 g/l of yeast extract and 20 g/l of Bacto-peptone, pH 6.7) containing 50 g/l of cellobiose, YPC50, was used for pre-cultivation of the cellobiose-fermenting strains (D-BT1m, D-CT1m, D-BT2m, and D-CT2m). YP medium containing 50 g/l of glucose, YPD50, was used for pre-cultivation of the glucose-fermenting strain expressing neither cellodextrin transporter nor cellobiose degrading enzyme (the parental strain containing empty plasmids, D-56 strain). Yeast cells at the exponential growth phase during the pre-cultivation were harvested and used for SSF. Seed cultivation and pre-cultivation were performed at 30 °C and 250 rpm.

*Lactobacillus fermentum* KCTC 3112 was used as the contaminant during SSF with contamination by LAB. De Man, Rogosa and Sharpe (MRS) medium (10 g/l of peptone, 8 g/l of meat extract, 4 g/l of yeast extract, 2 g/l of di-potassium hydrogen phosphate, 1 g/l of polysorbate 80, 1 g/l of di-ammonium hydrogen citrate, 5 g/l of sodium acetate, 0.2 g/l of magnesium sulfate, 0.04 g/l of manganese sulfate and 20 g/l of glucose, pH 6.5) was used for seed cultivation and pre-cultivation of *L. fermentum*. LAB cells at the exponential growth phase in the pre-cultivation were harvested and used for SSF with LAB contamination. Seed cultivation and pre-cultivation were performed at 30°C without agitation.

### Conditions for SSF of Cellulose

Avicel PH-101 (Sigma, USA) was used as the substrate for SSF. Celluclast 1.5L (Novozyme, Denmark) was used as the cellulase mixture for the saccharification of cellulose during SSF. Novozyme 188 (Novozyme) was used as the extracellular β-glucosidase for degradation of cellobiose to glucose during SSF with the parental strain containing empty plasmids. The inoculum size of yeast cells was adjusted to an optical density of 30, measured at 600 nm (OD600) and corresponding to 10.5 g/l of dry cell concentration, according to the condition determined in the previous studies [8-10].

SSF was carried out under three different conditions: (1) Micro-aerobic SSF was performed in 250-ml flasks filled with 50 ml of YP medium containing Avicel PH-101 (130 g/l) and Celluclast 1.5L [10 filter paper unit (FPU)/g cellulose], according to the conditions determined in the previous studies [8, 9]. The flasks were equipped with an airlock device (three-piece airlock with silicone stopper), releasing CO_2_ with minimized air-inflow. The temperature and agitation speed were maintained at 30°C and 100 rpm, respectively; (2) Anaerobic SSF was performed in 125-ml serum bottles filled with 25 ml of YP medium containing Avicel PH-101 (130 g/l) and Celluclast 1.5L (10 FPU/g cellulose). After purging the argon gas for 15 min, the serum bottles were tightly sealed with rubber caps, completely blocking the air-inflow. The bottles inoculated with yeast cells were maintained at 30°C and 100 rpm, respectively; (3) Anaerobic SSF contaminated by LAB was performed under the same conditions as anaerobic SSF, except for the co-inoculation of yeast and LAB cells. Novozyme 188 [5.4 cellobiase unit (CBU)/g cellulose] was used as the extracellular β-glucosidase for degradation of cellobiose to glucose during SSF with the parental yeast strain fermenting only glucose. The inoculum size of LAB cells for contamination was adjusted to OD600 of 3, which is equivalent to OD600 of 30 for yeast cells [20]. All SSF experiments were performed in triplicate.

### Analytical Methods

The concentrations of ethanol and lactate were determined by high-performance liquid chromatography (HPLC; Agilent Technologies 1200 Series, Agilent, USA) equipped with a refractive index (RI) detector using the Rezex ROA-Organic Acid H+ (8%) column (Phenomenex Inc., USA). The column was eluted with 0.005N of H_2_SO_4_ at a flow rate of 0.6 ml/min at 50°C.

## Results

### Performance of the Cellobiose-Fermenting *S. cerevisiae* Strains Expressing CDT-1 or CDT-2 during Micro-Aerobic SSF

In the previous studies, the D-CT1m strain exhibited as good ethanol production as that of the D-BT1m strain in the cellobiose fermentation and the SSF of cellulose [9, 12]. In another study, mutant CDT-2 was developed from wild-type CDT-2, whereby the D-CT2m strain fermented cellobiose as fast as the D-CT1m strain in the cellobiose fermentation [15]. These results suggested that the cellobiose-fermenting yeast strains expressing mutant CDT-2 (especially, D-CT2m) might demonstrate equivalent SSF performance to the cellobiose-fermenting yeast strains expressing mutant CDT-1 (both D-BT1m and D-CT1m). Therefore, to verify the ethanol production performance of the cellobiose-fermenting *S. cerevisiae* strains expressing mutant CDT-2 (D-BT2m and D-CT2m), SSF experiments with four cellobiose-fermenting yeast strains expressing either mutant CDT-1 or mutant CDT-2 were performed.

**Fig. 1:**
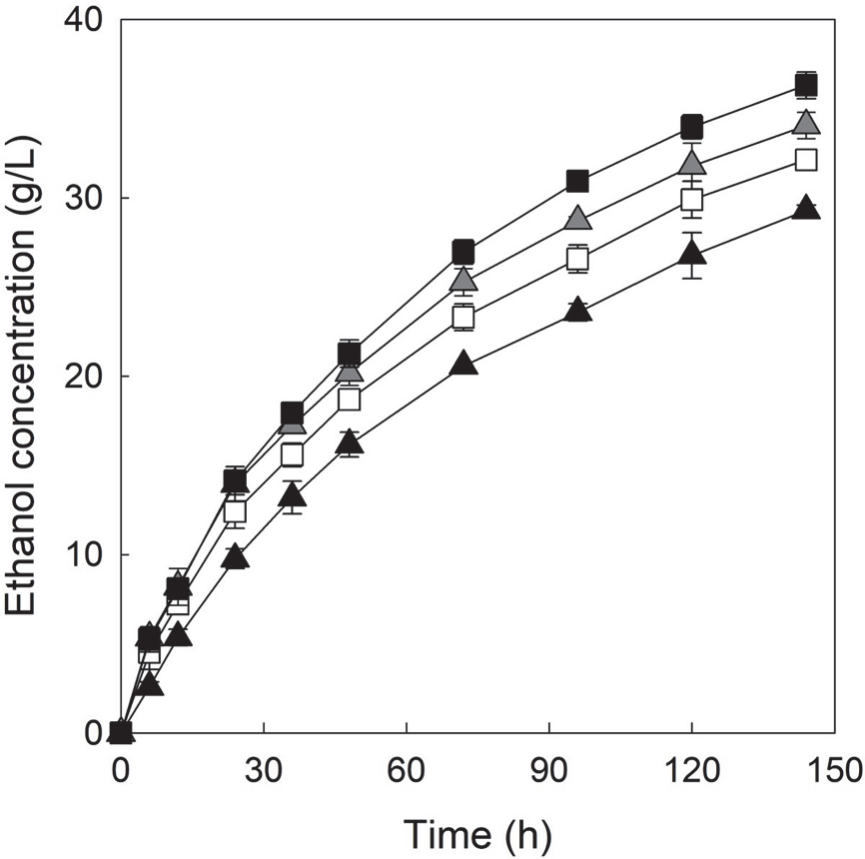
Ethanol production profiles during micro-aerobic SSF of 13% Avicel PH-101. SSF was performed by yeast cells with an OD600 value of 30 at 30°C and 100 rpm. Celluclast 1.5L (10 FPU/g cellulose) was used for the saccharification of cellulose. The yeast strains used for the SSF are as follows: D-BT1m (the hydrolytic *S. cerevisiae* expressing mutant CDT-1 and GH1-1; grey triangle, ▲); D-CT1m (the phosphorolytic *S. cerevisiae* expressing mutant CDT-1 and CBP; white square, □); DBT2m (the hydrolytic *S. cerevisiae* expressing mutant CDT-2 and GH1-1; black triangle, ▲); D-CT2m (the phosphorolytic *S. cerevisiae* expressing mutant CDT-2 and CBP; black square, ■). Ethanol concentration was measured in three independent experiments, and the symbols in the figure indicate average values with standard deviations.

Fig. 1 shows ethanol production profiles observed during micro-aerobic SSF of pure cellulose (Avicel PH-101) by four cellobiose-fermenting *S. cerevisiae* strains with mutant cellodextrin transporters.

When comparing ethanol production between two cellobiose-fermenting yeast strains expressing mutant CDT-2, D-CT2m showed considerably faster ethanol production than that of D-BT2m during the entire period of SSF. The difference in ethanol production between the two strains increased over time. Consequently, D-CT2m produced 24% more final ethanol than that produced by D-BT2m (36.3 g/l of ethanol by D-CT2m vs. 29.3 g/l of ethanol by D-BT2m), as shown in Table 2. Even though D-BT2m and D-CT2m expressed the same transporter, mutant CDT-2, the ethanol production by D-BT2m was considerably lower than that of D-CT2m. This is consistent with the previous study, which demonstrated a considerably lower ethanol yield and productivity by D-BT2m compared with those achieved by D-CT2m during the micro-aerobic cellobiose fermentation [15]. As yeast cells might generate sufficient energy in forms such as ATP by respiration under micro-aerobic conditions, the energetic benfit of the CBP-catalyzed reaction (saving one mole of ATP for degrading one mole of cellobiose compared with GH1-1-catalyzed reaction) might have little effect during SSF under micro-aerobic conditions [12, 15]. The enhancement of the ethanol production by D-CT2m over that of D-BT2m might be due to the 33%higher maximum specific growth rate (μ_max_) of D-CT2m for cellobiose than that of D-BT2m under micro-aerobic conditions, as shown in Fig. S2.

Meanwhile, when comparing ethanol production of two cellobiose-fermenting yeast strains expressing mutant CDT-1, D-BT1m produced 6% more final ethanol than that produced by D-CT1m during SSF (34.0 g/l of ethanol by D-BT1m vs.32.1 g/l of ethanol by D-CT1m), which is a consistent pattern with the results from the previous study [9].

Interestingly, when comparing the ethanol production by D-CT2m with those achieved by cellobiose-fermenting strains expressing mutant CDT-1, D-CT2m showed faster ethanol production than those of both D-BT1m and D-CT1m. Although D-CT2m produced a similar amount of ethanol compared with those of other strains (D-BT1m and D-CT1m) up to 12 h of SSF, subsequently, it produced more ethanol than that produced by other strains. The final concentration of ethanol produced by D-CT2m was 7% and 13% higher than those produced by D-BT1m and D-CT1m, respectively (Table 2). Similar to the CBP-catalyzed reaction, the energetic benefit of cellobiose transport based on the mutant CDT-2 (saving one mole of ATP for transporting one mole of cellobiose compared with the mutant CDT-1) might show little effect under the micro-aerobic condition [12, 15]. Therefore, the enhanced ethanol production by D-CT2m than those of both D-BT1m and D-CT1m might be attributed to the observation that D-CT2m showed a faster specific growth rate for cellobiose than those of other yeast strains expressing mutant CDT-1 under micro-aerobic conditions (Fig. S2). The final concentrations and yields of ethanol produced by cellobiose-fermenting *S. cerevisiae* expressing mutant cellodextrin transporters are summarized in Table 2.

### Performance of the Cellobiose-Fermenting *S. cerevisiae* Strains Expressing CDT-1 or CDT-2 during Anaerobic SSF

Compared with micro-aerobic SSF, anaerobic SSF was also performed to confirm whether there was a change in ethanol production by cellobiose-fermenting *S. cerevisiae* strains expressing mutant cellodextrin transporters when the oxygen supply was tightly restricted.

**Fig. 2:**
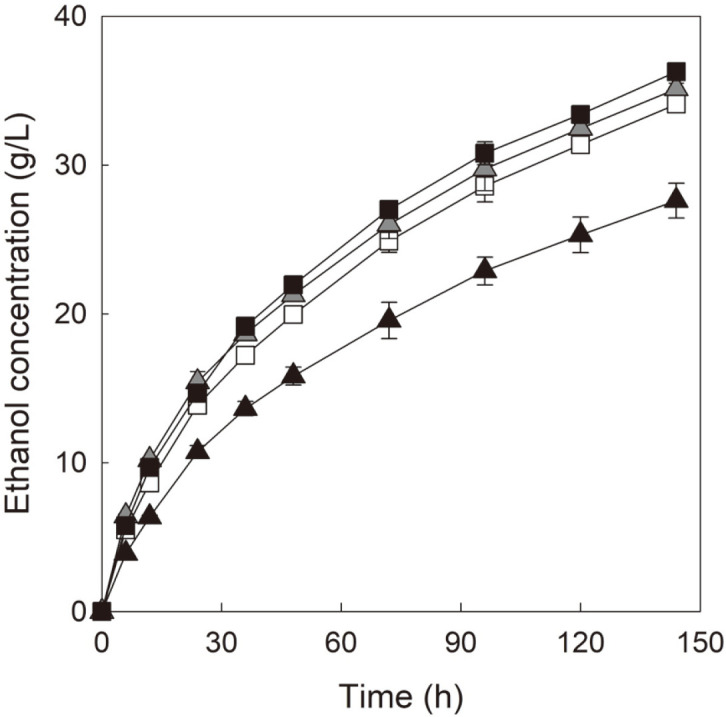
Ethanol production profiles during anaerobic SSF of 13% Avicel PH-101. SSF was performed by yeast cells with an OD600 value of 30 at 30°C and 100 rpm. Celluclast 1.5L (10 FPU/g cellulose) was used for the saccharification of cellulose. The yeast strains used for the SSF are as follows: D-BT1m (the hydrolytic *S. cerevisiae* expressing mutant CDT-1 and GH1-1; grey triangle, ▲); D-CT1m (the phosphorolytic *S. cerevisiae* expressing mutant CDT-1 and CBP; white square, □); DBT2m (the hydrolytic *S. cerevisiae* expressing mutant CDT-2 and GH1-1; black triangle, ▲); D-CT2m (the phosphorolytic *S. cerevisiae* expressing mutant CDT-2 and CBP; black square, ■). Ethanol concentration was measured in three independent experiments, and the symbols in the figure indicate average values with standard deviations.

Fig. 2 shows ethanol production profiles observed during anaerobic SSF of Avicel PH-101 by four cellobiose-fermenting *S. cerevisiae* strains with either mutant CDT-1 or mutant CDT-2.

When comparing ethanol production between two cellobiose-fermenting yeast strains expressing mutant CDT-2, D-CT2m showed a faster and higher ethanol production than that of D-BT2m throughout SSF, similar to that observed during micro-aerobic SSF. The gap of ethanol production between D-CT2m and D-BT2m expanded over time from an initial value of 3.3 g/l (at 12 h of SSF) to a final value of 8.7 g/l (at 144 h of SSF). As shown in Table 2, D-CT2m produced 36.3 g/l of ethanol, which was 31% higher than the amount of ethanol produced by D-BT2m. Notably, the difference in ethanol production between D-CT2m and D-BT2m during anaerobic SSF was larger than that observed during micro-aerobic SSF. These results suggest that the energetic benefit of D-CT2m based on CBP might become potent during anaerobic SSF involving limited energy generation by the yeast cells [12, 15]. Hence, compared with micro-aerobic SSF, D-CT2m seemed to produce a similar amount of ethanol during anaerobic SSF, whereas D-BT2m produced less amount of ethanol under SSF conditions with restricted aeration.

When comparing ethanol production between two cellobiose-fermenting strains expressing mutant CDT-1, D-BT1m showed a higher ethanol production than that of D-CT1m (35.1 g/l of ethanol by D-BT1m vs.34.1 g/l of ethanol by D-CT1m), similar to that observed during micro-aerobic SSF, consistent with the results from the previous studies [8, 9].

Similar to the results from micro-aerobic SSF, D-CT2m showed faster and higher ethanol production than those achieved by cellobiose-fermenting strains expressing mutant CDT-1. D-CT2m produced a similar level of ethanol compared with those of D-BT1m and D-CT1m during the early period of SSF (until 24 h of SSF). However, D-CT2m produced more ethanol than those produced by D-BT1m and D-CT1m from the middle of SSF (after 72 h of SSF). The final concentration of ethanol produced by D-CT2m was 3% and 6% higher than those produced by D-BT1m and D-CT1m, respectively (Table 2). As mentioned above, enhanced ethanol production by D-CT2m than those by other strains expressing mutant CDT-1 might be attributed to the energy-saving effects based on both mutant CDT-2 and CBP [12, 15]. When considering that the gap of ethanol production between D-CT2m and D-CT1m was higher than that between D-CT2m and D-BT1m, it might be reasonable to state that the energy-saving effect of mutant CDT-2 was greater than that of CBP in regards to the ethanol production by D-CT2m. The final concentrations and yields of ethanol produced by cellobiose-fermenting *S. cerevisiae* expressing mutant cellodextrin transporters are summarized in Table 2.

### Performance of the Phosphorolytic Cellobiose-Fermenting *S. cerevisiae* Expressing CDT-2 during Anaerobic SSF Contaminated by LAB

The results from micro-aerobic and anaerobic SSF of cellulose suggest that the D-CT2m showed superior ethanol production performance than those of other three cellobiose-fermenting *S. cerevisiae* strains (D-BT1m, D-CT1m and D-BT2m), regardless of SSF conditions.

Meanwhile, bacterial contamination during ethanol fermentation is a serious problem reducing ethanol yield and productivity of the biofuels production from starch, sugarcane, and cellulosic biomass [21-23]; the main bacterial contaminants are gram-positive bacteria, including LAB [20-24]. Among various LAB, *L. fermentum* has been found as the most dominant contaminant in bioethanol production [20, 23, 24]. Since *L. fermentum* cannot utilize cellobiose [25], it was expected that cellobiose-fermenting yeast strain would be advantageous for mitigating the reduction of ethanol yield and productivity in SSF contaminated by *L. fermentum*. Consequently, SSF of cellulose contaminated by *L. fermentum* was performed to verify whether the D-CT2m could also show better SSF performance than those of other yeast strains, such as the traditional glucose-fermenting *S. cerevisiae* supplemented with extracellular β-glucosidase (D-56+188), and the hydrolytic cellobiose-fermenting *S. cerevisiae* expressing mutant CDT-1 (D-BT1m).

**Fig. 3:**
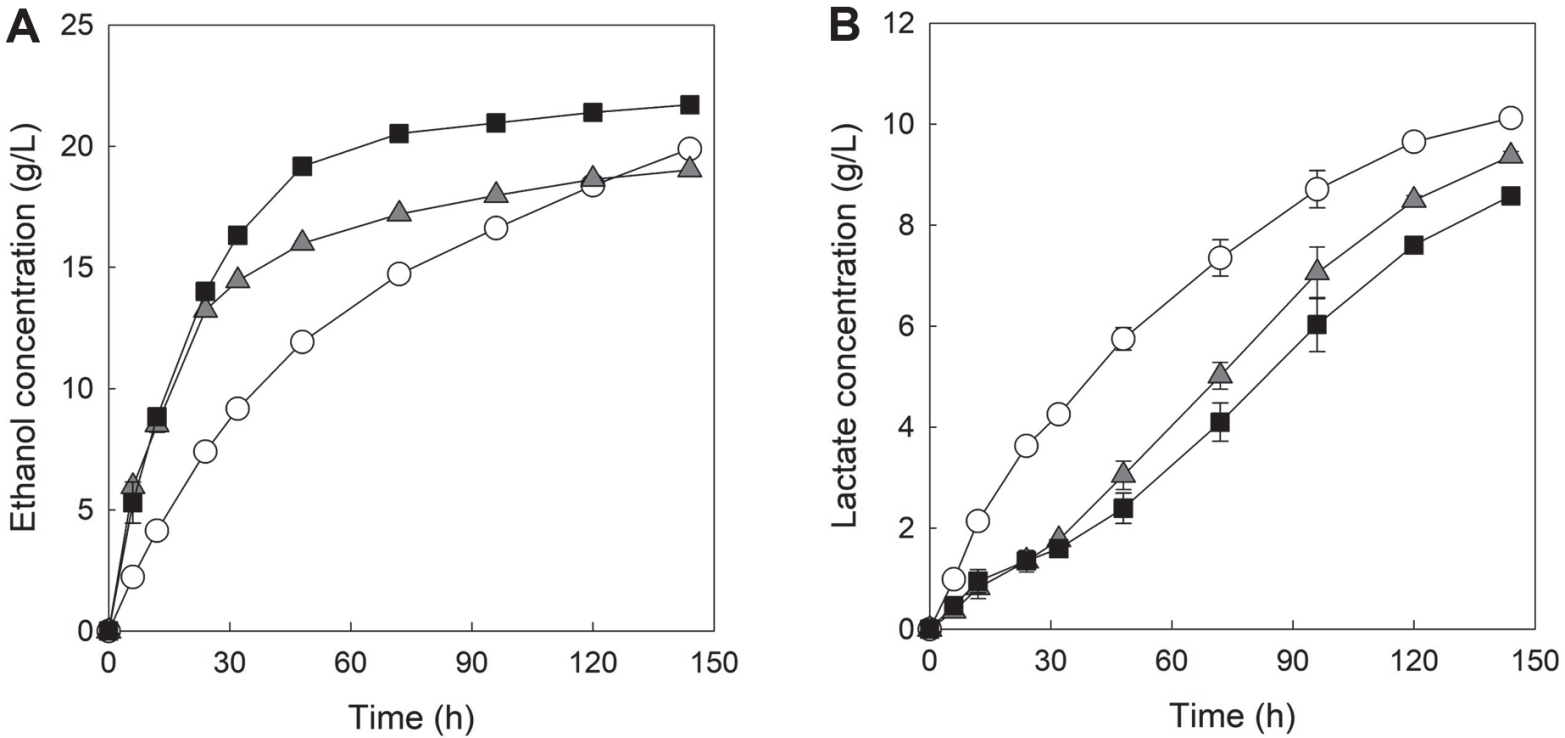
Profiles of ethanol production (A) and lactate formation (B) during anaerobic SSF of 13% Avicel PH- 101 contaminated by lactic acid bacteria (LAB). SSF was performed by yeast cells with an OD600 value of 30 at 30°C and 100 rpm. For contamination of SSF by LAB, *L. fermentum* cells with an OD600 value of 3 were co-inoculated with yeast cells. The yeast strains used for the SSF are as follows: D-56+188 (the parental glucose-fermenting *S. cerevisiae* with extracellular β-glucosidase; white circle, ○); D-BT1m (the hydrolytic *S. cerevisiae* expressing mutant CDT-1 and GH1-1; grey triangle, ▲); D-CT2m (the phosphorolytic *S. cerevisiae* expressing mutant CDT-2 and CBP; black square, ■). Celluclast 1.5L (10 FPU/g cellulose) was used for saccharification of cellulose. In SSF with D-56+188, Novozyme 188 (5.4 CBU/g cellulose) was added along with Celluclast 1.5L for degradation of cellobiose to glucose. Ethanol and lactate concentrations were measured in three independent experiments, and the symbols in the figure indicate average values with standard deviations.

Fig. 3 shows the profiles of ethanol production by three yeast strains (D-BT1m, D-CT2m, and D-56+188) and lactate formation by *L. fermentum* during anaerobic SSF of Avicel PH-101 contaminated by the same number of LAB cells as the yeast cells.

In the case of LAB-contaminated SSF performed with D-56+188 strain, glucose can be primarily released from the saccharification of cellulose upon addition of Celluclast 1.5L along with β-glucosidase. The D-56+188 strain might be unable to efficiently produce ethanol during the entire period of SSF because the glucose-fermenting *S. cerevisiae* might have to compete against *L. fermentum* for glucose uptake from the beginning of SSF [20, 25]. Hence, the pattern of ethanol production by D-56+188 was similar to the pattern of lactate formation by *L. fermentum* (like logarithmic curve shape), and the ratio for ethanol production to lactate formation remained constant throughout the entire duration of SSF (ratio for ethanol to lactate ≈ 2.0 g/g). The final concentrations of ethanol and lactate produced in LAB-contaminated SSF with D-56+188 were 19.9 g/l and 10.1 g/l, respectively, as shown in Table 2.

In the case of LAB-contaminated SSF with D-BT1m strain, cellobiose can be primarily released from the saccharification of cellulose upon addition of Celluclast 1.5L without β-glucosidase. This suggests that the D-BT1m strain might produce ethanol efficiently at the early stage of SSF because the cellobiose-fermenting *S. cerevisiae* might not have to compete fiercely against *L. fermentum* for glucose at the beginning of SSF. However, as SSF progressed, the release of a small amount of glucose from cellulose by Celluclast 1.5L might promote the gradual activation of LAB cells growth, which might have caused several stressful and inhibitory effects on the growth and sugar metabolism of yeast cells [21, 22]. Therefore, efficient production of ethanol by D-BT1m seems to have been prevented after an early period of SSF (32 h of SSF). As the results, the pattern of ethanol production by D-BT1m exhibited hyperbolic curve shape while the pattern of lactate formation by *L. fermentum* was observed in a similar shape of a sigmoidal curve. In particular, the ratio for ethanol production to lactate formation of D-BT1m was initially high but rapidly decreased after 32 h of SSF (8.2 g/g at 32 h, 2.5 g/g at 96 h and 2.0 g/g at 144 h). Consequently, the hydrolytic cellobiose-fermenting *S. cerevisiae* strain expressing mutant CDT-1 showed an even lower production of final ethanol than that produced by the parental *S. cerevisiae* with extracellular β-glucosidase at the end of SSF (19.0 g/l of ethanol and 9.4 g/l of lactate in SSF with D-BT1m) as shown in Table 2.

Remarkably, D-CT2m showed the fastest ethanol production among the strains tested during SSF contaminated by LAB. The energy-saving effects of D-CT2m based on the mutant CDT-2 and CBP seems to have been maximized under these harsh conditions [12, 15], including no supply of oxygen and competition of cell growth against LAB contamination. Similar to the D-BT1m strain, D-CT2m might not have to compete fiercely against *L. fermentum* for glucose at the beginning of SSF. However, LAB cells might grow progressively over time, which might have prevented efficient production of ethanol by D-CT2m from the middle stage of SSF (48 h of SSF). As the results, similar to LAB-contaminated SSF with D-BT1m strain, ethanol production by D-CT2m and lactate formation by *L. fermentum* showed hyperbolic and sigmoidal curve shapes, respectively. Nonetheless, due to the energy-saving effects based on mutant CDT-2 and CBP, D-CT2m might have produced more ethanol than that produced by D-BT1m even after the middle stage of SSF. Ethanol production by D-CT2m was slowed down after 48 h of SSF, which was 16 h later than time for slowdown of ethanol production observed in SSF by D-BT1m, and the ratio for ethanol production to lactate formation of D-CT2m was slowly decreased compared with that of D-BT1m (ratio for ethanol to lactate in D-CT2m: 8.0 g/g at 48 h, 3.5 g/g at 96 h and 2.5 g/g at 144 h). Hence, SSF performed with D-CT2m showed the highest production of ethanol and the lowest formation of lactate among all SSF experiments contaminated by LAB (21.7 g/l of ethanol and 8.6 g/l of lactate in SSF with D-CT2m). Particularly, ethanol production by D-CT2m was 9% and 14% higher than those by D-56+188 and D-BT1m, respectively (Table 2).

As Fig. S3 shows that D-BT1m and D-CT2m produced similar amounts of ethanol during anaerobic SSF in the presence of 5 g/l of lactate, compared with anaerobic SSF in the absence of lactate, it can be proposed that LAB itself (including the formation of several extracellular metabolites along with cell growth) would be the main factor inhibiting ethanol production of the cellobiose-fermenting *S. cerevisiae* strains rather than lactate accumulation during SSF contaminated by *L. fermentum* [21, 22], although weak organic acid such as lactate has been known to show inhibitory effect on yeast cell growth [26]. Based on the results from LAB-contaminated SSF with the glucose- or cellobiose-fermenting yeast strains, the inhibitory effects by LAB on ethanol production could be significantly alleviated during SSF performed by D-CT2m compared with SSF performed by other yeast strains. The final concentrations and yields of ethanol produced by the glucose-fermenting *S. cerevisiae* with extracellular β-glucosidase and the cellobiose-fermenting *S. cerevisiae* expressing mutant cellodextrin transporters are summarized in Table 2.

## Discussion 

In the previous studies, irrespective of the type of intracellular cellobiose degrading enzymes, the cellobiose-fermenting yeast strains with the mutant CDT-1 (energy-dependent active cellodextrin transporter) exhibiting enhanced cellobiose transporting activity showed similar ethanol production performance during the SSF of cellulose [8, 9], suggesting that the cellobiose transporting activity of the cellodextrin transporter would be the key factor affecting the efficiency of the cellobiose-fermenting yeast during the SSF of cellulose.

Meanwhile, the cellobiose transporting capacity of the mutant CDT-2 (energy-independent cellodextrin facilitator) was improved to a level comparable to that of the mutant CDT-1 [15]. However, the transporting capacity of the mutant CDT-2 for cellodextrin, such as cellotriose and cellotetraose, was still lower than that of mutant CDT-1 [12, 15]. In addition, CBP has considerably lower activity to cellodextrin than that to cellobiose, whereas GH1-1 has an as good activity to cellodextrin as that to cellobiose [11, 12]. Considering that the intermediate cellodextrin can also be released with the main product, cellobiose, from the saccharification of cellulose by cellulase enzymes during SSF, it was doubtful that the D-CT2m strain could demonstrate better ethanol production performance than those of other cellobiose-fermenting *S. cerevisiae* strains, such as D-BT1m and D-CT1m, during the SSF of cellulose. However, D-CT2m showed superior ethanol production performance than those of other cellobiose-fermenting *S. cerevisiae* strains during the SSF of cellulose.

During the micro-aerobic SSF of cellulose shown in Fig. 1, D-CT2m showed a higher ethanol production than that of D-BT2m, which was consistent with our expectations because D-BT2m had shown approximately two-fold slower ethanol production due to the significant accumulation of extracellular cellodextrin during cellobiose fermentation compared with that of D-CT2m, as reported in the previous study [15]. It is interesting to note that D-CT2m produced more ethanol than those produced by D-BT1m and D-CT1m, which was in contrast to our expectations because energy-saving effects of D-CT2m based on mutant CDT-2 and CBP might be substantially suppressed under micro-aerobic conditions where the yeast cells might generate sufficient energy without tight limitation of oxygen supply. The higher ethanol production by D-CT2m than those of other yeast strains might be attributed to the enhanced μ_max_ of D-CT2m for cellobiose, which was 12%, 4% and 33% higher than those of other yeast strains expressing mutant cellodextrin transporters for cellobiose, respectively (μ_max_ of D-BT1m for cellobiose: 0.25 h^−1^; μ_max_ of D-CT1m for cellobiose: 0.27 h^−1^; μ_max_ of D-BT2m for cellobiose: 0.21 h^−1^; μ_max_ of D-CT2m for cellobiose: 0.28 h^−1^) [8, 9], as observed in Fig. S2.

During the anaerobic SSF of cellulose shown in Fig. 2, D-CT2m showed considerably higher ethanol production than that of D-BT2m, which might be because D-BT2m has fewer advantage in terms of energy-saving than that of D-CT2m. It might also because D-BT2m shows significantly slower growth rate than that of D-CT2m for cellobiose as mentioned above. Similar to the results from micro-aerobic SSF, D-CT2m showed better ethanol production than those of other yeast strains expressing mutant CDT-1, which was consistent with our expectations because energy-saving effects of D-CT2m based on mutant CDT-2 and CBP might become prominent under anaerobic conditions [12, 15]. Considering that D-CT1m produced less ethanol than that produced by D-BT1m during anaerobic SSF, the energy-saving effects shown by D-CT2m may be primarily attributed to the mutant CDT-2 rather than CBP.

During anaerobic SSF contaminated by *L. fermentum* shown in Fig. 3, D-BT1m produced ethanol faster than D-56+188 until the late stage of SSF, and D-CT2m showed the fastest ethanol production compared with other yeast strains until the end of SSF. These results suggest that the cellobiose-fermenting yeast would provide benefits, such as alleviating the reduction of ethanol yield and productivity during SSF of cellulose contaminated by LAB. In particular, D-CT2m showed the lowest lactate formation along with the highest ethanol production among the tested strains, suggesting that D-CT2m could compete most effectively against *L. fermentum* through the energy-saving effects by mutant CDT-2 and CBP during SSF contaminated by LAB. However, *L. fermentum* is known as a heterofermentative lactic acid bacterium that produces 0.51 g/l of ethanol when producing 1 g/l of lactate [27]. This indicates that the actual concentration of ethanol produced by the cellobiose-fermenting yeast strains (when calculated by excluding the concentration of ethanol that is estimated to be produced by *L. fermentum*) was reduced by more than 50% during SSF contaminated by LAB compared with that observed during anaerobic SSF without LAB contamination (35.1 g/l by D-BT1m and 36.3 g/l by D-CT2m during anaerobic SSF vs. 14.2 g/l by D-BT1m and 17.3 g/l by D-CT2m during anaerobic SSF with *L. fermentum* contamination). Consequently, bacterial contamination can be a serious problem that reduces ethanol yield and productivity in biofuel production [21-23]. Furthermore, utilization of the cellobiose-fermenting yeast may be limited only to certain types of SSF, such as SSF contaminated by LAB that cannot metabolize cellobiose like *L. fermentum*. In the case of LAB capable of metabolizing cellobiose well, such as *Lactobacillus plantarum* [28], the cellobiose-fermenting yeast did not effectively produce ethanol at all during SSF contaminated by *L. plantarum* (production of ethanol less than 5 g/l, data not shown), suggesting that other strategies should also be conceived to minimize the problems caused by LAB contamination. In addition to the utilization of the cellobiose-fermenting yeast for the SSF of cellulose, the utilization of several enzymes triggering LAB cell death by breaking down the peptidoglycan of LAB may be beneficial [29-31].

In this study, it was observed that the D-CT2m performed better during the SSF of cellulose than other yeast strains such as the cellobiose-fermenting *S. cerevisiae* expressing mutant CDT-1 (D-BT1m and D-CT1m). It was also observed that the D-CT2m strain showed the best ethanol production and the lowest lactate formation during SSF contaminated by *L. fermentum*, compared with those of other strains, such as the D-BT1m and the glucose-fermenting *S. cerevisiae* with extracellular β-glucosidase (D-56+188). Such enhanced ethanol production by the D-CT2m strain may be attributed to the energetic benefits by CBP as well as mutant CDT-2. Based on the results from the current study, it can be suggested that the cellobiose-fermenting yeast employing a cellobiose metabolic pathway consuming a lower amount of energy would be useful in the SSF of cellulosic biomass for the production of biofuels and biochemicals.

## Supplemental Materials

Supplementary data for this paper are available on-line only at http://jmb.or.kr.

## Figures and Tables

**Table 1:** Plasmids and *S. cerevisiae* strains used in this study.

Plasmids and strains	Relevant features	Reference
Plasmids		
pRS425PGK	*LEU2*, P_PGK_-MCS-T_CYC_, 2μ origin, Amp^r^	[11]
pRS425-gh1-1	*LEU2*, P_PGK_-*gh1-1*-T_CYC_, 2μ origin, Amp^r^	[11]
pRS425-CBP	*LEU2,* P_PGK_-*CBP*-T_CYC_, 2μ origin, Amp^r^	[12]
pRS426PGK	*URA3*, P_PGK_-MCS-T_CYC_, 2μ origin, Amp^r^	[11]
pRS426-cdt1 (F213L)	*URA3*, P_PGK_-*cdt1* (F213L)-T_CYC_, 2μ origin, Amp^r^	[12]
pRS426-cdt2 (N306I)	*URA3*, P_PGK_-*cdt2* (N306I)-T_CYC_, 2μ origin, Amp^r^	[15]
Strains		
D452-2	*MATα*, *leu2*, *his3*, *ura3* and *can1*	[19]
D-56	D452-2/pRS425PGK /pRS426PGK	[8]
D-56+188	D-56 with extracellular β-glucosidase	[8]
D-BT1m	D452-2/pRS425-gh1-1/pRS426-cdt1 (F213L)	[12]
D-CT1m	D452-2/pRS425-CBP/pRS426-cdt1 (F213L)	[12]
D-BT2m	D452-2/pRS425-gh1-1/pRS426-cdt2 (N306I)	[15]
D-CT2m	D452-2/pRS425-CBP/pRS426-cdt2 (N306I)	[15]

**Table 2:** Summary of the results from the SSF of cellulose performed by *S. cerevisiae* strains fermenting cellobiose.

Culture conditions	Strains	Final ethanol (g/l)	Final lactate (g/l)	Ethanol yield* from cellulose (g/g)
Micro-aerobic, 13% Avicel, Celluclast 1.5L (10 FPU/g cellulose)	D-BT1m	34.0 ± 0.74	-	0.29
	D-CT1m	32.1 ± 0.56	-	0.27
	D-BT2m	29.3 ± 0.31	-	0.25
	D-CT2m	36.3 ± 0.75	-	0.31
Anaerobic, 13% Avicel, Celluclast 1.5L (10 FPU/g cellulose)	D-BT1m	35.1 ± 0.38	-	0.30
	D-CT1m	34.1 ± 0.29	-	0.29
	D-BT2m	27.6 ± 1.17	-	0.23
	D-CT2m	36.3 ± 0.40	-	0.31
Anaerobic, 13% Avicel, Celluclast 1.5L (10 FPU/g cellulose), *L. fermentum* ((OD600 ~3))	D-56+188	19.9 ± 0.08	10.1 ± 0.13	0.17
	D-BT1m	19.0 ± 0.03	9.4 ± 0.09	0.16
	D-CT2m	21.7 ± 0.21	8.6 ± 0.09	0.18

*Since Avicel exhibits 9% water content, as shown in the previous study [8], the ethanol yield from 13% Avicel was calculated based on the final ethanol concentration divided by 118.3 g/l of actual Avicel concentration. In SSF with D-56+188, Novozyme 188 (5.4 CBU/g cellulose) was added along with Celluclast 1.5L for degradation of cellobiose to glucose.
