# Supplementary material for: Effects of Engineered Saccharomyces cerevisiae Fermenting Cellobiose through Low-Energy-Consuming Phosphorolytic Pathway in Simultaneous Saccharification and Fermentation
Source: J Microbiol Biotechnol. 2021 Dec 9;32(1):117–25. doi: 10.4014/jmb.2111.11047 (PMC9628822; doi:10.4014/jmb.2111.11047)
Supplement: Supplementary file 1 [file jmb-32-1-117-supple.pdf]

J. Microbiol. Biotechnol.
https://doi.org/10.4014/jmb.2111.11047

Supplementary Materials

Comparison of cellobiose metabolic pathways in engineered S. cerevisiae strains expressing cellodextrin transporters and intracellular cellobiose degrading enzymes

To develop engineered *S. cerevisiae* strains capable of fermenting cellobiose, the cellobiose metabolic pathway, composed of cellodextrin transporters and intracellular cellobiose degrading enzymes, has been introduced into *S. cerevisiae* [11-13].

Cellodextrin transporters identified from *N. crassa* are classified into two types depending on whether energy is required for cellobiose transport or not. CDT-1 is a proton symporter requiring the consumption of ATP by ATPase to export the protons that enter the cell along with cellobiose [11, 14]. CDT-2 is a facilitator transporting cellobiose depending on the concentration gradient of extracellular cellobiose [11, 14]. Consequently, CDT-1 spends one mole of ATP to transport one mole of cellobiose, while CDT-2 spends no ATP for cellobiose transport.

Intracellular cellobiose degrading enzymes identified from *N. crassa* or *S. degradans* are classified into two types based on the degree of energy consumption. Intracellular β-glucosidase (GH1-1) from *N. crassa* is involved in the hydrolysis of cellobiose (cellobiose → 2 glucose), which requires the consumption of two moles of ATP by hexokinases converting glucose to glucose-6-phosphate to initiate glycolysis from cellobiose [11]. Cellobiose phosphorylase (CBP) from *S. degradans* is involved in the phosphorolysis of cellobiose (cellobiose → glucose + glucose-1-phosphate), which requires only one mole of ATP consumption by hexokinases to initiate glycolysis from cellobiose due to phosphoglucomutase isomerizing glucose-1-phosphate to glucose-6-phosphate without ATP [12]. Consequently, cellobiose hydrolysis by GH1-1 spends

two moles of ATP to initiate glycolysis from one mole of cellobiose, whereas cellobiose phosphorolysis by CBP spends one mole of ATP for glycolysis from one mole of cellobiose.

According to the combination of cellodextrin transporters and intracellular cellobiose degrading enzymes, four types of the cellobiose-fermenting *S. cerevisiae* strains have been developed [11, 12, 14, 15]; the amounts of energy required for each strain to transport cellobiose and start glycolysis are as follow: 3 moles of ATP for D-BT1 strain (*S. cerevisiae* expressing CDT-1 and GH1-1); 2 moles of ATP for D-CT1 strain (*S. cerevisiae* expressing CDT-1 and CBP); 2 moles of ATP for D-BT2 strain (*S. cerevisiae* expressing CDT-2 and GH1-1); 1 mole of ATP for D-CT2 strain (*S. cerevisiae* expressing CDT-2 and CBP). Fig. S1 illustrates the differences in energy consumption between each cellobiose-fermenting *S. cerevisiae* strain according to the types of cellobiose transport and intracellular degradation.

Determination of growth kinetic parameters of the cellobiose-fermenting S. cerevisiae strains expressing mutant CDT-2

Growth kinetic parameters, such as Monod constant (K_S) and maximum specific growth rate (μ_{max}), of the cellobiose-fermenting *S. cerevisiae* strains expressing mutant CDT-2 (D-BT2m and D-CT2m strains) under cellobiose conditions were determined as follows. Yeast cells at the exponential growth phase in pre-cultivation were harvested, washed twice with sterilized water, and inoculated into 50 mL of minimal (SC) medium with different initial concentrations of cellobiose (0 to 2 g/L) at an initial OD600 of 0.05. The culture was carried out at 30°C and 100 rpm, and the OD values of the culture broth were checked every 2 h. The growth kinetic parameters of D-BT2m and D-CT2m strains were determined by non-linear regression of the

plots for the specific growth rates over various concentrations of the initial cellobiose. All cell culture experiments were performed in triplicate.

Fig. S2 compares the profiles of specific growth rates of the cellobiose-fermenting *S. cerevisiae* strains expressing either mutant CDT-1 or mutant CDT-2 (D-BT1m, D-CT1m, D-BT2m and D-CT2m) for various initial cellobiose concentrations. The μ_{max} and K_s values of D-BT1m and D-CT1m for cellobiose have already been determined in the previous studies (0.25 h^{-1} and 0.06 g/L of cellobiose for D-BT1m; 0.27 h^{-1} and 0.06 g/L of cellobiose for D-CT1m) [8, 9]. In the current study, the μ_{max} and K_s values of D-BT2m for cellobiose were determined to be 0.21 h^{-1} and 0.07 g/L of cellobiose, respectively. The μ_{max} and K_s values of D-CT2m for cellobiose were determined to be 0.28 h^{-1} and 0.07 g/L of cellobiose, respectively.

Performance of the cellobiose-fermenting S. cerevisiae strains expressing CDT-1 or CDT-2 during anaerobic SSF in the presence of lactate

Ethanol productions of the cellobiose-fermenting *S. cerevisiae* strains expressing either mutant CDT-1 or mutant CDT-2 (D-BT1m and D-CT2m strains) and the glucose-fermenting *S. cerevisiae* strain (D-56+188 strain) were compared during the anaerobic SSF of cellulose in the presence of lactate. As the D-BT1m and D-CT2m strains showed a significant slowdown in ethanol production after 72 h (the time when lactate was accumulated over 5 g/L) of anaerobic SSF contaminated by *L. fermentum*, anaerobic SSF with lactate was performed in 125-mL serum bottles filled with 25 mL of YP medium containing Avicel PH-101 (130 g/L), lactate (5 g/L) and Celluclast 1.5L (10 FPU/g cellulose) with or without Novozyme 188 (5.4 CBU/g cellulose). SSF experiments were performed in triplicate.

Fig. S3 compares ethanol production profiles of D-BT1m, D-CT2m and D-56+188 strains observed during anaerobic SSF of Avicel PH-101 with 5 g/L of lactate. Similar to anaerobic SSF without lactate, D-CT2m showed the fastest and the highest ethanol production among the three yeast strains. Although D-BT1m exhibited an inferior ethanol production performance to that of D-CT2m, it still exhibited a faster and higher ethanol production than that of D-56+188, consistent pattern with the results from the previous studies [8, 9]. Final concentrations of ethanol produced by D-CT2m, D-BT1m and D-56+188 were 35.9 g/L, 33.7 g/L and 31.9 g/L, respectively. Because ethanol productions by D-BT1m and D-CT2m in SSF with lactate were not significantly reduced when compared with those of SSF without lactate, the inhibitory effect of lactate accumulation on ethanol production of the cellobiose-fermenting *S. cerevisiae* is considered to be insignificant during SSF contaminated by lactic acid bacteria.

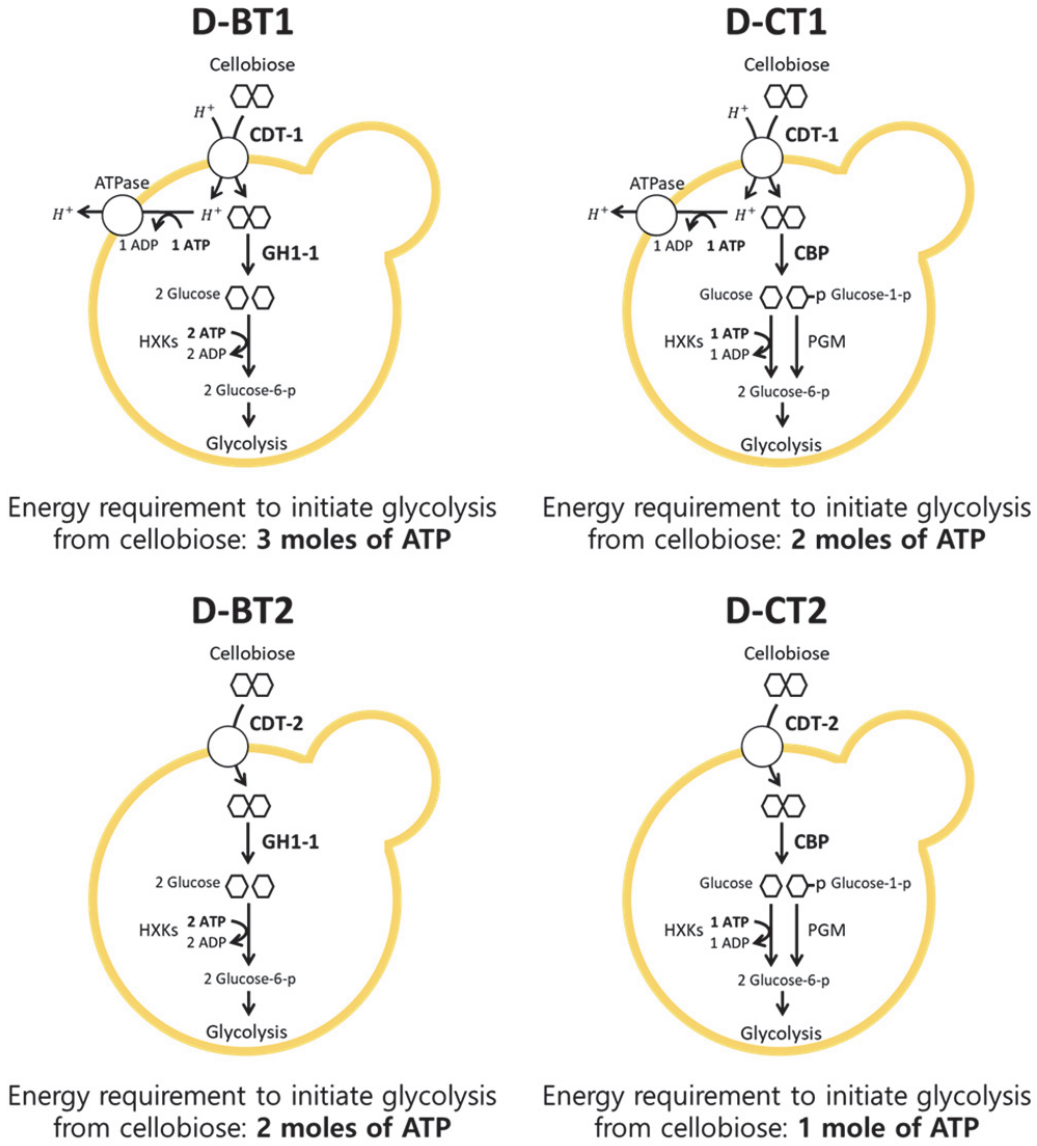

Fig. S1. Comparison of cellobiose metabolic pathways in the cellobiose-fermenting *S. cerevisiae* strains based on the types of cellobiose transporters and intracellular cellobiose degrading enzymes. Extracellular cellobiose is transported into the cell by either CDT-1 or CDT-2. The transported cellobiose is degraded by either intracellular β-glucosidase (GH1-1) or cellobiose phosphorylase (CBP). The degraded products of cellobiose, two glucose or one glucose with one glucose-1-phosphate, are converted to glucose-6-phosphate by hexokinases (HXKs) and

phosphoglucomutase (PGM). The description of each yeast strain is as follows: D-BT1 (the hydrolytic *S. cerevisiae* expressing CDT-1 and GH1-1); D-CT1 (the phosphorolytic *S. cerevisiae* expressing CDT-1 and CBP); D-BT2 (the hydrolytic *S. cerevisiae* expressing CDT-2 and GH1-1); D-CT2 (the phosphorolytic *S. cerevisiae* expressing CDT-2 and CBP).

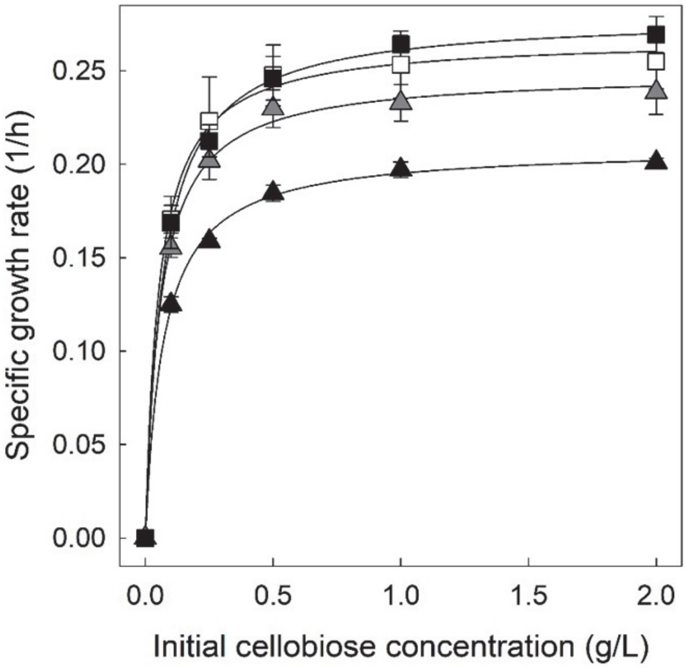

Fig. S2. Comparison of the specific growth rates of the cellobiose-fermenting *S. cerevisiae* strains grown in minimal (SC) medium with various initial concentrations of cellobiose (0 to 2 g/L) under micro-aerobic conditions (yeast cells with initial OD600 of 0.05, 30 °C and 100 rpm). The description of each yeast strain is as follows: D-BT1m (the hydrolytic *S. cerevisiae* expressing mutant CDT-1 and GH1-1; grey triangle, ▲); D-CT1m (the phosphorolytic *S. cerevisiae* expressing mutant CDT-1 and CBP; white square, □); D-BT2m (the hydrolytic *S. cerevisiae* expressing mutant CDT-2 and GH1-1; black triangle, ▲); D-CT2m (the phosphorolytic *S. cerevisiae* expressing mutant CDT-2 and CBP; black square, ■). Cell cultivation experiments in the current study were performed in triplicate, and the symbols in the figure show average values with standard deviations. The profiles of specific growth rate of D-BT2m and D-CT2m obtained in the current study were compared with the profiles of specific growth rate of D-BT1m and D-CT1m published in the previous studies [8, 9].

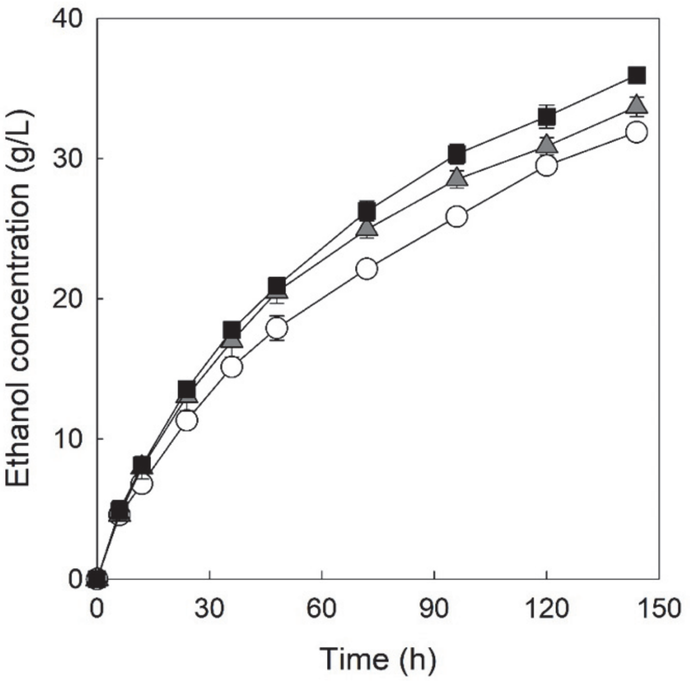

Fig. S3. Ethanol production profiles during anaerobic SSF of 13% Avicel PH-101 containing 5 g/L of lactate. SSF was performed by yeast cells with an OD600 value of 30 at 30 °C and 100 rpm. Celluclast 1.5L (10 FPU/g cellulose) was used for the saccharification of cellulose. The yeast strains used for the SSF are as follows: D-56+188 (the parental glucose-fermenting *S. cerevisiae* with extracellular β-glucosidase; white circle, ○); D-BT1m (the hydrolytic *S. cerevisiae* expressing mutant CDT-1 and GH1-1; grey triangle, ▲); D-CT2m (the phosphorolytic *S. cerevisiae* expressing mutant CDT-2 and CBP; black square, ■). Celluclast 1.5L (10 FPU/g cellulose) was used for saccharification of cellulose. In SSF with D-56+188, Novozyme 188 (5.4 CBU/g cellulose) was added along with Celluclast 1.5L for degradation of cellobiose to glucose. Ethanol concentration was measured in three independent experiments, and the symbols in the figure indicate average values with standard deviations.
